# Supplementary figures and images for: The deficiency of myelin in the mutant taiep rat induces a differential immune response related to protection from the human parasite Trichinella spiralis
Source: PLoS One. 2020 Aug 20;15(8):e0231803. doi: 10.1371/journal.pone.0231803 (PMC7444528; doi:10.1371/journal.pone.0231803)

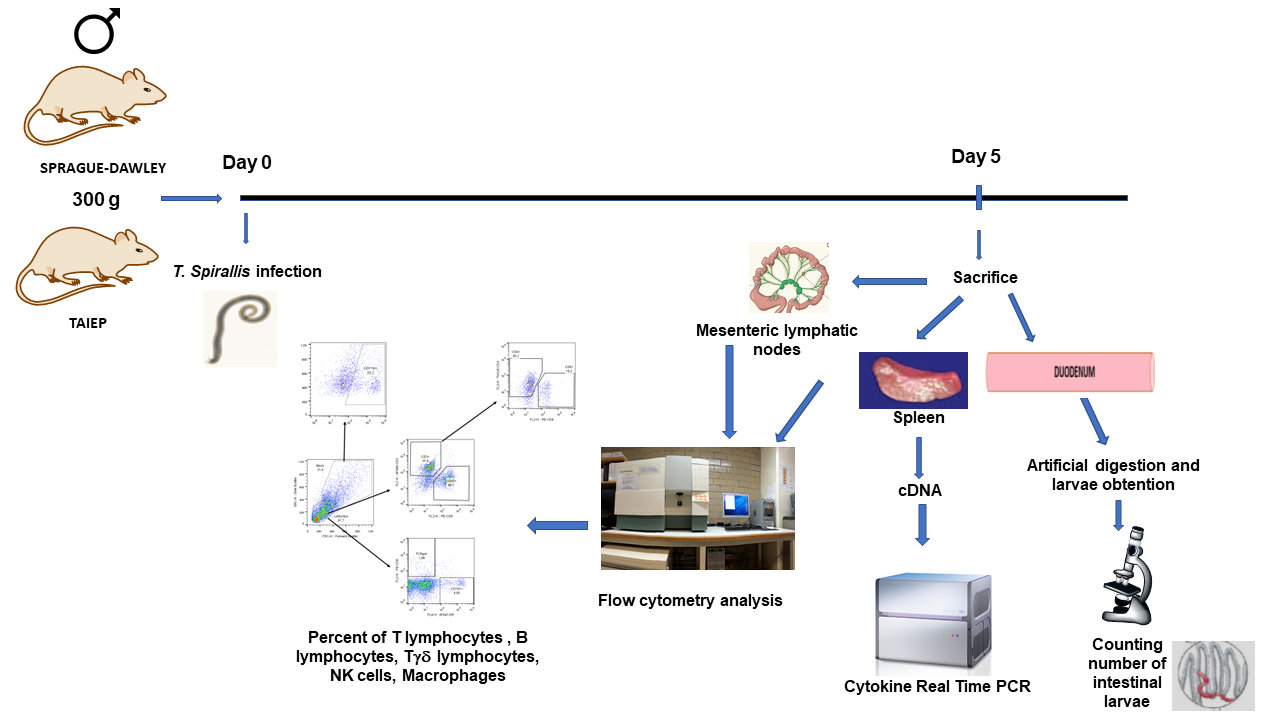

Supplement: S1 Fig — (TIF) [file pone.0231803.s001.tif]
